# Which Method Best Predicts Postoperative Complications: Deep Learning, Machine Learning, or Conventional Logistic Regression?

**DOI:** 10.1002/ags3.70145

**Published:** 2025-12-04

**Authors:** Ryosuke Fukuyo, Masanori Tokunaga, Hiroyuki Yamamoto, Hideki Ueno, Yusuke Kinugasa

**Affiliations:** ^1^ Department of Gastrointestinal Surgery Institute of Science Tokyo Tokyo Japan; ^2^ Database Committee The Japanese Society of Gastroenterological Surgery Tokyo Japan; ^3^ Department of Healthcare Quality Assessment Graduate School of Medicine, the University of Tokyo Tokyo Japan; ^4^ Department of Surgery National Defense Medical College Saitama Japan

**Keywords:** deep learning, postoperative complications, prediction models, surgical complications

## Abstract

Accurate prediction of postoperative complications is critical in surgical care. Recently, deep learning has gained attention and has been applied to various predictive models and image recognition tasks, and researchers are attempting to apply this technology in medicine. This review compares logistic regression, machine learning, and deep learning models used in gastroenterological surgery. There are some studies reporting predictive models with large databases. Among these studies, some studies showed that deep learning outperformed other models, but others reported random forests or gradient boosting methods, a type of machine learning, performed better than the other methods including deep learning. On the other hand, applying image or time‐series data are reported to increase the prediction accuracy of postoperative morbidity/mortality, despite relatively small sample sizes. While deep learning shows potential, especially with image and time‐series data, it often underperforms on tabular clinical datasets such as current National Clinical Database (NCD). We discuss the limitations of deep learning in term of its “black‐box” nature and highlight the need for integrating complex data types to improve model accuracy and interpretability. Incorporating multimodal inputs may enable deep learning to outperform conventional methods and better support clinical decision‐making.

## Graphical abstract

Deep learning has shown promise in predicting postoperative complications, particularly when using image or time‐series data. However, on tabular clinical data such as the NCD, it often underperforms compared to conventional machine learning. Integrating multimodal data may enhance predictive accuracy and interpretability in surgical care.

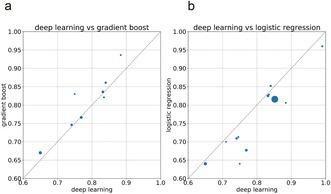

## 1 Introduction

Recently, minimally invasive surgeries for cancer have gained attention among gastroenterological surgeons due to their favorable outcomes compared to open surgery [1]. These outcomes include faster recovery, reduced bleeding, and fewer postoperative morbidities. However, the incidence of postoperative morbidity and mortality varies depending on the surgical procedure and remains nonzero, prompting surgeons to seek alternative methods. Therefore, it is indispensable to evaluate surgical risk, including postoperative morbidity and mortality, before surgery.

Logistic regression models have been established to predict postoperative morbidity and mortality before surgery. Additionally, odds ratios are commonly used to estimate the impact of each covariate on the outcome variable. Although logistic models are relatively simple compared to deep learning models, they allow for straightforward interpretation of covariate effects, the development of postoperative complications involves complex mechanisms. Thus, a deeper understanding of statistical models used in medical data analysis, including those predicting postoperative complications, is essential.

In recent years, deep learning has gained attention and has been applied to various predictive models and image recognition tasks. Deep learning is a type of machine learning that uses neural networks with a multilayered structure, designed to mimic human neurons and enable predictions and judgments [2]. Deep learning is a subfield of machine learning; however, due to its unique architecture and broad applicability, it is often regarded as a distinct domain separate from conventional machine learning methods. Researchers are attempting to apply this technology in medicine, hoping it will enhance our understanding by effectively processing large volumes of complex data previously difficult to analyze. While deep learning has produced remarkable results in various fields and is revolutionizing society, it is important to recognize its limitations to ensure its appropriate and effective use in medicine [3].

This paper reviews existing risk calculation models for gastroenterological surgeries and discusses the potential advantages and limitations of deep learning compared with traditional logistic regression and other machine learning approaches.

## 2 Conventional (Non‐Machine Learning) Surgical Risk Models

Traditional risk prediction in surgery has primarily relied on statistical models such as logistic regression or scoring systems derived from multivariable analyses. Various complication prediction models have been developed since the 20th century, some of which are shown in Table 1 [4, 5, 6, 7, 8, 9, 10, 11, 12, 13]. Among these, Charlson Comorbidity Score, reported in 1987, is the earliest and includes 21 covariates [4]. The Charlson Comorbidity Score is reportedly associated with early survival outcomes, with a reported ROC‐AUC of 0.76 [5]. The Physiological and Operative Severity Score for the Enumeration of Mortality and Morbidity (POSSUM) and Portsmouth‐POSSUM (P‐POSSUM) were developed by Copeland and Prytherch to predict morbidity and mortality in general surgery patients, with an ROC‐AUC of 0.84 [6, 7, 8]. The Surgical Apgar Score is a simple risk calculation model comprising only three covariates: intraoperative blood loss, blood pressure, and heart rate [10]. It enables complication prediction immediately after surgery without burden, but the ROC‐AUC of 0.71 is lower than that of the aforementioned models [11]. Preoperative Score to Predict Postoperative Mortality (POSPOM) is an easy‐to‐use, rule‐based scoring system that predicts postoperative mortality with high accuracy (ROC‐AUC 0.93) [14].

**TABLE 1:** Conventional surgical risk model.

Year	Author	Scoring system	Country	No. of covariates	C‐statistics
1987	Charlson ME	Charlson Comorbidity Index	United States	21	0.76
1998	Prytherch DR	Severity Score for the Enumeration of Mortality and Morbidity (POSSUM/P‐POSSUM)	United Kingdom	12	0.84
2001	Kamath PS	Model for End‐Stage Liver Disease (MELD)	United States	4	0.87
2007	Gawande	Surgical Apger Score	United States	3	0.71
2011	Dalton	Risc Quantification Index	United States	3	0.91
2012	Nashef SA	EuroSCOREII	United Kingdom	17	0.81
2016	Le Manach	Preoperative Score to Predict Post operative Mortality (POSPOM)	France	29	0.93

Although these risk models are considered useful, their predictive accuracy declines significantly when applied to patients with different clinical backgrounds. For example, POSPOM was developed in France with an ROC‐AUC of 0.93, but an external validation study in Germany reported a lower ROC‐AUC of 0.771 [15]. Therefore, it is indispensable to develop more accurate and generalized predictive models applicable to diverse patient populations.

## 3 Risk Calculation Models Based on NCD

In Japan, various surgical risk calculators have been developed, with recent attention focused on models based on the National Clinical Database (NCD). Among these, those in the field of gastrointestinal surgery are presented in Table 2 [16, 17, 18, 19, 20, 21, 22, 23, 24, 25, 26, 27, 28, 29, 30, 31, 32, 33, 34, 35, 36, 37, 38]. The NCD is a web‐based registry system that collects data through standardized questionnaires. While the details vary slightly between versions, the 2019 version includes approximately 300 questionnaire items, including those that differ depending on the surgical procedure. The NCD‐based risk calculators, typically developed using logistic regression, use 11–47 covariates. Theoretically these models can be applied to any patients in a Japanese hospital involved in NCD. Various models have been developed for different surgical procedures and endpoints, and their predictive accuracy varies accordingly. Models predicting mortality tend to be more accurate than those predicting morbidity, likely because mortality represents a more clearly defined endpoint. The NCD risk calculator predicts postoperative mortality with a c‐statistic of 0.71–0.90, the highest for right hemicolectomy [21]. In contrast, morbidity prediction models show broader variation in accuracy, being 0.50–0.88, depending on the type of surgery and complications [31, 32, 33, 34, 35, 36, 37]. These calculators are currently available on the NCD website and are widely used by Japanese surgeons to estimate surgical risks before surgery.

**TABLE 2:** NCD‐based surgical risk model.

Year	Author	Procedure	Outcome	No. of covariates	c‐statistics
2014	Watanabe M	Total gastrectomy	Mortality	18	0.81
2014	Kimura W	Pancreoduodenectomy	Mortality	13	0.73
2014	Takeuchi H	Esophagectomy	Mortality	12	0.77
2014	Kenjo A	Hepatectomy	Mortality	23	0.71
2014	Matsubara N	Low anterior resection	Mortality	17	0.77
2014	Kobayashi H	Right hemicolectomy	Mortality	16	0.90
2015	Kurita N	Distal gastrectomy	Mortality	21	0.79
2015	Nakagoe T	Acute diffuse peritonitis	Mortality	26	0.85
2019	Marubashi S	Liver transplants	Mortality	7	0.74
2023	Kawai K	Low anterior resection	Mortality	26	0.79
2023	Sasaki A	Esophagectomy	Mortality	25	0.71
2024	Orimo T	Hepatectomy	Mortality	36	0.84
2024	Sato T	Acute diffuse peritonitis	Mortality	47	0.86
2025	Mizuma M	Pancreoduodenectomy	Mortality	28	0.76
2015	Saze Z	Acute diffuse peritonitis	Complications	18–19*	0.69–0.85**
2016	Yokoo H	Hepatectomy	Complications	20–27*	0.63‐0.80**
2017	Kikuchi H	Total gastrectomy	Complications	10–25*	0.63–0.80**
2017	Kunisaki C	Distal gastrectomy	Complications	13–25*	0.70–0.83**
2017	Watanabe T	Low anterior resection	Complications	11–20*	0.593–0.799**
2017	Aoki S	Pancreoduodenectomy	Complications	21–2*	0.70‐0.71**
2018	Yoshida T	Right hemicolectomy	Complications	15–21*	0.63–0.88**
2019	Ohkura Y	Esophagectomy	Complications	14–21*	0.53‐0.64**

* No. of covariates varies depending on the target complication.

** Accuracy varies depending on the target complication.

## 4 Risk Calculation Models With Deep Learning

Recently, web‐based nationwide databases in Japan and other countries have enabled researchers to work with large datasets with hundreds of thousands of patients. Around 2020, devices and software used for deep learning became publicly available, allowing researchers to conduct advanced analyses without the need for large‐scale data centers. As a result, many attempts have been made to establish more accurate risk models using deep learning that surpass the performance of conventional logistic regression models [39, 40, 41, 42, 43, 44, 45, 46, 47, 48, 49, 50, 51, 52, 53, 54, 55, 56, 57, 58, 59, 60, 61, 62, 63, 64, 65].

**FIGURE 1:**
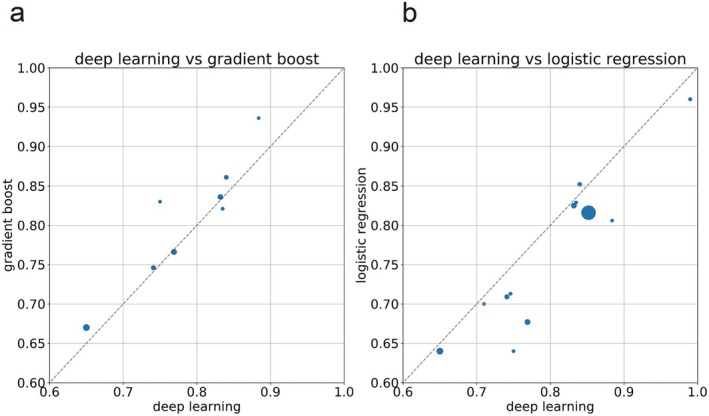
(a) Scatter plot of C‐statistics for Deep learning vs. Gradient boosting in complication prediction models. (b) Deep learning vs. Logistic regression. The size of each point reflects the number of cases reported.

**FIGURE 2:**
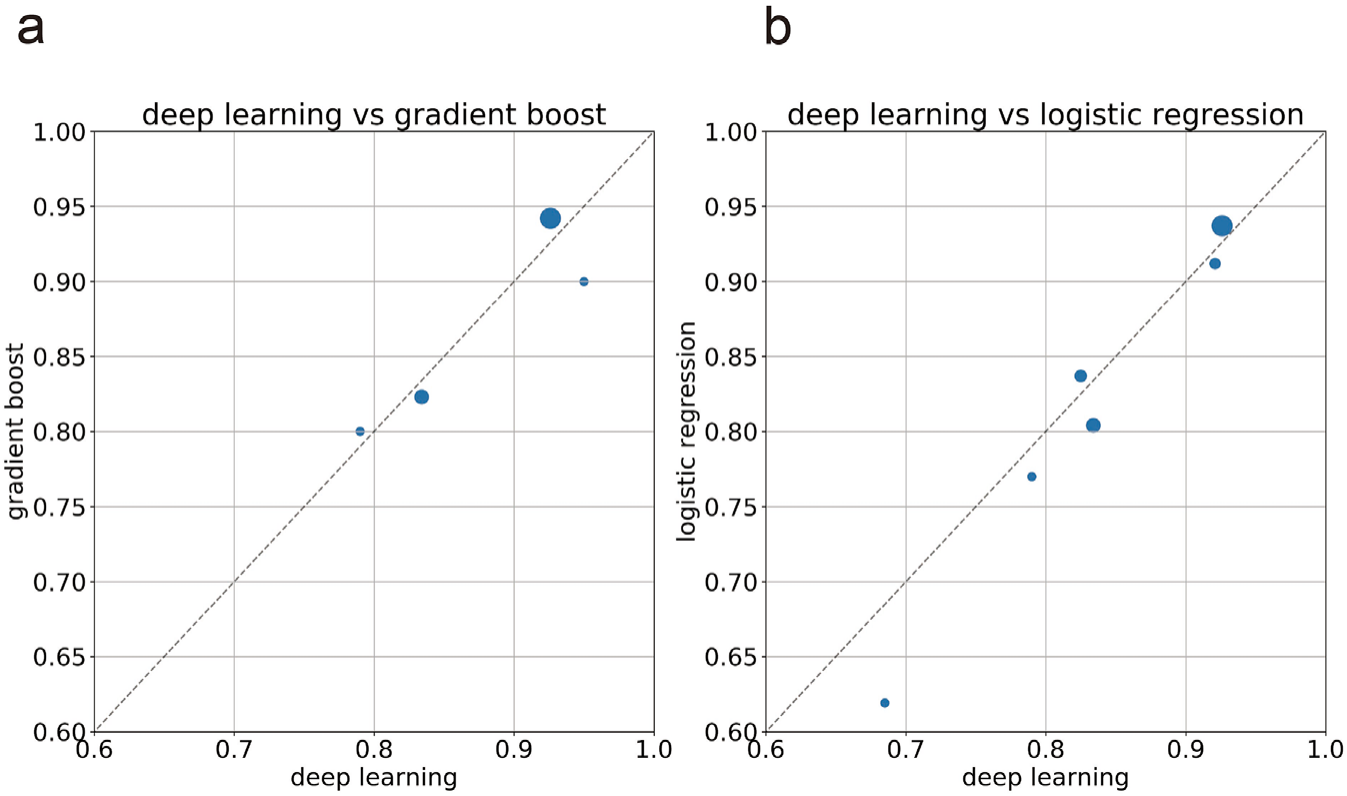
(a) Scatter plot of C‐statistics for Deep learning vs. Gradient boosting in mortality prediction models. (b) Deep learning vs. Logistic regression. The size of each point reflects the number of cases reported.

A PubMed search in July 2025 using the terms “deep learning” and “postoperative complications” or “postoperative mortality” identified 502 and 127 articles, respectively, at the time of search (Figure S1). Abstracts were screened by R.F., and articles reporting deep learning methods and c‐statistics were included. Exclusion criteria included studies unrelated to gastrointestinal surgery, those without descriptions of machine learning or deep learning methods, review articles, editorials, and studies without relevant outcomes. Following full‐text assessment. 17 reports related to complications (Figure 1, Table 3) [29, 30, 31, 32, 33, 34, 35, 36, 37, 38, 39, 40, 41, 42, 43, 44, 45, 46, 47, 48, 49, 50, 51, 52, 53, 54, 55] and 10 reports related to mortality (Figure 2, Table 4) [56, 57, 58, 59, 60, 61, 62, 63, 64, 65] were extracted.

**TABLE 3:** Deep learning or machine learning models to predict complications.

					C‐statistics			
Year	Author	Surgery	Objective	No. of patient	Deep learning	Random forest	Gradient Boost	Logistic Regression
2021	Bonde A	Any	Any complication	58,81 881	0.874			
2023	Mamlook REA	Any	Surgical site infection	28,82 526	0.852	0.838		0.816
2021	Nudel J	Bariatric surgery	Deep vein thrombosis	4,36 807	0.65		0.67	0.64
2023	Chen KA	Colorectal cancer	Surgical site infection	2,75 152	0.769	0.764	0.766	0.677
2025	Min JW	Non cardiac surgery	Acute Kidney Injury	2,39 267	0.832	0.813	0.836	0.825
2023	Hsu JL	Bariatric surgery	Gastrointestinal bleeding	1,59 959	0.741	0.764	0.746	0.709
2021	Xue B	Any	Pneumonia	1,11 888	0.84	0.844	0.861	0.852
2020	Cao Y	Bariatric surgery	Any complication	44 061	0.55			
2023	Adiyeke E	Any	Acute Kidney Injury	42 906	0.99	0.95		0.96
2024	Bonde M	Pancreoduodenectomy	Any complication	31 728	0.669	0.635		
2022	Chen KA	Pancreoduodenectomy	Pancreatic fistura	23 333	0.746			0.713
2025	Xu M	Neurosurgery	Pulmonary complication	12 657	0.835	0.821	0.821	0.829
2022	Na JE	Endoscopic submucosal dissection	Post‐ESD bleeding	5629	0.71			0.7
2023	Wei R	Rectal cancer	Any complication	4313	0.75	0.77	0.83	0.64
2021	Chen C	Liver transplant	Pneumonia	786		0.781	0.794	0.68
2023	Fukuyo R	Gastric cancer	Any complication	727	0.7			
2024	Liu X	Colorectal cancer	Deep vein thrombosis	429	0.884	0.973	0.936	0.806

**TABLE 4:** Deep learning or machine learning models to predict mortality.

					C‐statics			
Year	Author	Surgery	Objective	No of patient	Deep learning	Random forest	Gradient Boost	Logistic Regression
2022	Lee SW	Non cardiac surgery	Mortality	4,54 404	0.926	0.885	0.942	0.937
2021	Orfanoudaki A	Mitral valve surgery	Mortality	3,83 550		0.77	0.826	0.825
2023	Allou N	Cardiac surgery	Mortality	1,65 640	0.834	0.824	0.823	0.804
2019	Fritz BA	Any	Mortality	95 970	0.825	0.848		0.837
2022	Harris AHS	Hip fracture surgery	Mortality	82 168	0.76			
2021	Lee CK	Any	Mortality	59 985	0.921			0.912
2025	Fukuyo R	Total gastrectomy	Mortality	14 980	0.74			
2021	Cho J	Any	Mortality	3817	0.79	0.75	0.8	0.77
2023	Wise E	Duodenal switch	Mortality	2907	0.685			0.619
2020	Jalali A	Pediatric heart surgery	Mortality	549	0.95	0.84	0.9	0.55

For postoperative morbidity, Bonde et al. and Mamlook et al. used large databases (with millions of cases) and achieved good prediction accuracy, with c‐statistics of 0.87 and 0.85, respectively (Table 3) [39, 40]. The accuracy of complication prediction is higher when the target complication is more specific, such as renal dysfunction or deep venous thrombosis, probably because clearly defined targets may reduce labeling noise and facilitate the extraction of features strongly associated with the outcome, thereby enhancing learning efficiency. Otherwise, publication bias may have contributed; easily predicted complications tend to have higher prediction accuracy, likely to be published. Fourteen of the seventeen reviewed studies compared their predictive values with those of other machine learning methods. In seven studies, deep learning outperformed other models, while in the remaining seven, random forests or gradient boosting methods performed better. For example, Hsu et al. used a dataset of approximately 160 000 cases to predict gastrointestinal bleeding after bariatric surgery, where random forest performed better than deep learning [44]. Similarly, Xue et al. used data from approximately 110 000 cases to create a model for predicting postoperative pneumonia prognosis, reporting that gradient boosting as performing better [45]. In contrast, Chen et al. reported that deep learning outperformed other models in predicting surgical site infections following colorectal surgery [42]. In studies with relatively small dataset with hundreds of cases, deep learning and machine learning appear to perform very well over conventional logistic regression [53, 54, 55]. This result is very interesting because deep learning and machine learning are generally said to over‐learn and lose accuracy when the number of data is limited. However, publication bias will need to be considered in these relatively small datasets.

Similar trends were observed mortality prediction models (Table 4) [56, 57, 58, 59, 60, 61, 62, 63, 64, 65]. Among nine studies, eight reported c‐statistics comparing deep learning with other methods; four favored deep learning, while the remaining four found other models superior. The latter included the largest study by Lee et al. and Orfanoudaki et al. [56, 57]

## 5 Discussion

Various methods are used in the development of complication prediction models. Recently, deep learning has attracted researchers, as neural network‐based models with multilayered structure are expected to outperform conventional models. However, despite its use in many fields, deep learning is not always the most effective method for modeling tabular medical data.

In Japan, there are attempts to use machine learning and deep learning to create risk models, similar to the NCD risk calculator mentioned above. Sakamoto et al. used machine learning to create a model for predicting suture failure after rectal resection [66]. Fukuyo et al. used deep learning to create a model for predicting death after total gastrectomy [62]. The purpose of these studies was to outperform conventional logistic regression analysis by using new methods, but none of them showed any improvement in accuracy. Similar attempts have been made in the United States. Liu et al. used National Surgical Quality Improvement Program (NSQIP) patient records to create a predictive model with extreme gradient boosting [67]. Compared to the regression model, the prediction accuracy increased, but the difference was limited. Although it is not meaningful to simply compare c‐statistics than conventional methods because these analyses were performed on different patient backgrounds, at least the expected results were not obtained by changing the method alone. Since expanding tabular data alone are insufficient to achieve a remarkable improvement in accuracy, the use of additional modalities such as image or audio data would be considered. However, integrating these into the existing database may raise technical and ethical concern, requiring careful discussion, and these complex models may compromise interpretability.

Deep learning is often described as a “black box” because the process of prediction is very complex, which makes it nearly impossible for surgeons to interpret how individual covariate influence outcomes. To solve this black‐box nature, various studies have been conducted. Explainable AI (XAI) such as SAHP or LIME was invented to improve transparency and trust in deep learning models; however, several challenges remain. The explanations provided by XAI methods are often approximate and may vary across techniques, raising concerns about reliability. In addition, interpretation of feature importance or heatmaps requires expertise and may lead to misunderstanding. Standardized evaluation criteria and regulatory frameworks for XAI in clinical applications are still lacking. However, the decision‐making process is important to surgical risk prediction, as understanding which variables influence outcomes can help guide therapy choice to reduce morbidity and mortality. Considering the “black‐box” characteristics and computational complexity of deep learning, its predictive accuracy must significantly surpass that of conventional models or provide clear interpretability and clinical applicability in order to be accepted as a standard prediction model in clinical practice.

One reason deep learning may be less effective is likely the lack of relationships between adjacent variables in tabular data. When applying deep learning models to image data, the contrast with the surroundings is crucial, then the color or density of a particular pixel itself. Additionally, in table data, relationships between neighboring data are minimal, rendering deep learning less effective. Furthermore, the data number is often limited in the medical field, leading to concerns of reduced generalizability due to overlearning. In these cases, the model over‐fits the training data and does not fit unknown data appropriately, including an extra‐validation set. To overcome these limitations, incorporating more complex data types, such as images, should be considered to surpass other models like random forests, gradient boosting, or logistic regression.

Deep learning shows particular strengths in areas involving complex data, such as image and time‐series analysis, which should be involved in future surgical risk prediction models.

### 5.1 Image Analysis, Time Series Data Analysis

Images, audio, and video data are typically larger than tabular data. These data contain interdependent features, allowing deep learning models, such as convolutional neural networks (CNN), to efficiently extract local patterns. These methods are currently being investigated for applications in intraoperative navigation and complication prediction based on CT imaging.

Similarly, time‐series data such as speech waveforms can now be analyzed with unprecedented accuracy using deep learning‐based pattern recognition.

### 5.2 Natural Language Processing

In natural language, the meaning of a word or a sentence often depends on the surrounding context. Both data processing and recognizing the relationships between the data and the context are important. Recurrent Neural Network (RNN) was developed to capture these relationships, and more recently, transformer‐based models such as GPT have achieved more accurate natural language processing. These models have enabled applications such as automatic information extraction from electronic medical records, which could contribute to future risk predictive models.

In gastroenterological surgery, studies using intraoperative image data are also under investigation. Ryu et al. developed a surgical support system to highlight the composition of connective tissue during surgery using image recognition [68, 69]. Khojah et al. reported a system for intraoperative ureteral highlighting, and Igaki et al. developed an automatic surgical skill assessment system by analyzing surgical videos with deep learning [70, 71]. Cai et al. used preoperative MRI to predict the need for multiple staples during rectal resection [72]. These examples illustrate the potential of intraoperative imaging to enhance real‐time surgical support.

In addition to patient factors, surgeon and facility‐related factors have been identified as important determinants of postoperative complications [44, 45, 49, 51]. The technical aspects of surgery play a crucial role, making their evaluation essential. Surgical video assessment offers a promising avenue for directly evaluating surgeon skill, and further advances in this field are anticipated. Incorporating assessments of surgeon skill and facility performance may further enhance predictive accuracy. However, changes in complication probabilities attributable to surgeons should be interpreted cautiously from a perspective of causality.

Applying image or time‐series data is reported to increase the prediction accuracy of postoperative morbidity/mortality, despite small sample sizes. For example, Jeong et al. used preoperative MRI to predict postoperative liver failure after hepatectomy, and Mu et al. developed a model for pancreatic leak prediction based on preoperative CT [73, 74]. Park et al. reported a model predicting postoperative renal dysfunction using intraoperative vital sign data [75]. These studies demonstrate the potential of combining deep learning with multimodal data to improve perioperative management.

To build effective deep learning‐based surgical risk predictive models, it is essential to integrate complex data types such as images, audio, and video. Considering that surgeons evaluate surgical risk using these multimodal inputs, deep learning models with more complex data are essential to outperform human judgment and traditional models. NCD is Japan's largest database, and due to its characteristics, it is difficult to dramatically increase the number of cases. An international multicenter study could be considered to enhance data volume; however, the resulting heterogeneity in patient characteristics may limit the reliability and generalizability. Furthermore, the establishment of such an international database would raise considerable ethical and legal concerns, making this approach difficult to implement. Alternatively, incorporating image or audio data may improve predictive accuracy, but technical and ethical problems are anticipated when integrating such data into the database. It is necessary to carefully consider whether the inclusion of audio or image data in an anonymized database would be justified. In addition, the volume of such data would be extremely large, making data management and operation more challenging. The methodology for integrating new types of data should first be evaluated in small‐scale studies prior to large‐scale implementation. Therefore, to achieve further improvements in predictive accuracy, various methodological innovations will likely be required to overcome these barriers.

## 6 Conclusion

There is a growing need for more accurate postoperative complication prediction models, and deep learning represents a promising approach to achieve this. However, current deep learning models that rely solely on tabular data (e.g., current dataset from the NCD) do not consistently outperform conventional models. To improve the accuracy of deep learning‐based prediction models, it is indispensable to incorporate more complex parameters. Including images and videos is a plausible option; however, more basic studies are necessary for future implementation.

## Author Contributions

**Ryosuke Fukuyo:** methodology, data curation, investigation, formal analysis, visualization, project administration, writing – original draft, software, writing – review and editing. **Masanori Tokunaga:** conceptualization, methodology, writing – review and editing, supervision, project administration, validation, investigation. **Hiroyuki Yamamoto:** conceptualization, writing – review and editing, supervision, validation. **Hideki Ueno:** conceptualization, writing – review and editing, supervision. **Yusuke Kinugasa:** conceptualization, supervision, writing – review and editing.

## Funding

The authors have nothing to report.

## Ethics Statement

The authors have nothing to report.

## Conflicts of Interest

Yusuke Kinugasa and Hideki Ueno are editorial members of Annals of Gastroenterological Surgery. Hiroyuki Yamamoto is affiliated with the Department of Healthcare Quality Assessment at the University of Tokyo, which is a social collaboration department supported by grants from the National Clinical Database, Intuitive Surgical Sarl, Johnson & Johnson K.K., and Nipro Co.

## Supporting information


**Figure S1:** Flowchart of research. We searched using the terms “deep learning” and “postoperative complications” or “postoperative mortality.” Abstracts were screened by R.F., and articles reporting deep learning methods were extracted.
