# Supplementary material for: Which Method Best Predicts Postoperative Complications: Deep Learning, Machine Learning, or Conventional Logistic Regression?
Source: Ann Gastroenterol Surg. 2025 Dec 4;10(3):653–62. doi: 10.1002/ags3.70145 (PMC13178273; doi:10.1002/ags3.70145)
Supplement: Supplementary file 1 — Figure S1: Flowchart of research. We searched using the terms “deep learning” and “postoperative complications” or “postoperative mortality.” Abstracts were screened by R.F., and articles reporting deep learning methods were extracted. [file AGS3-10-653-s001.pptx]

## Slide 1
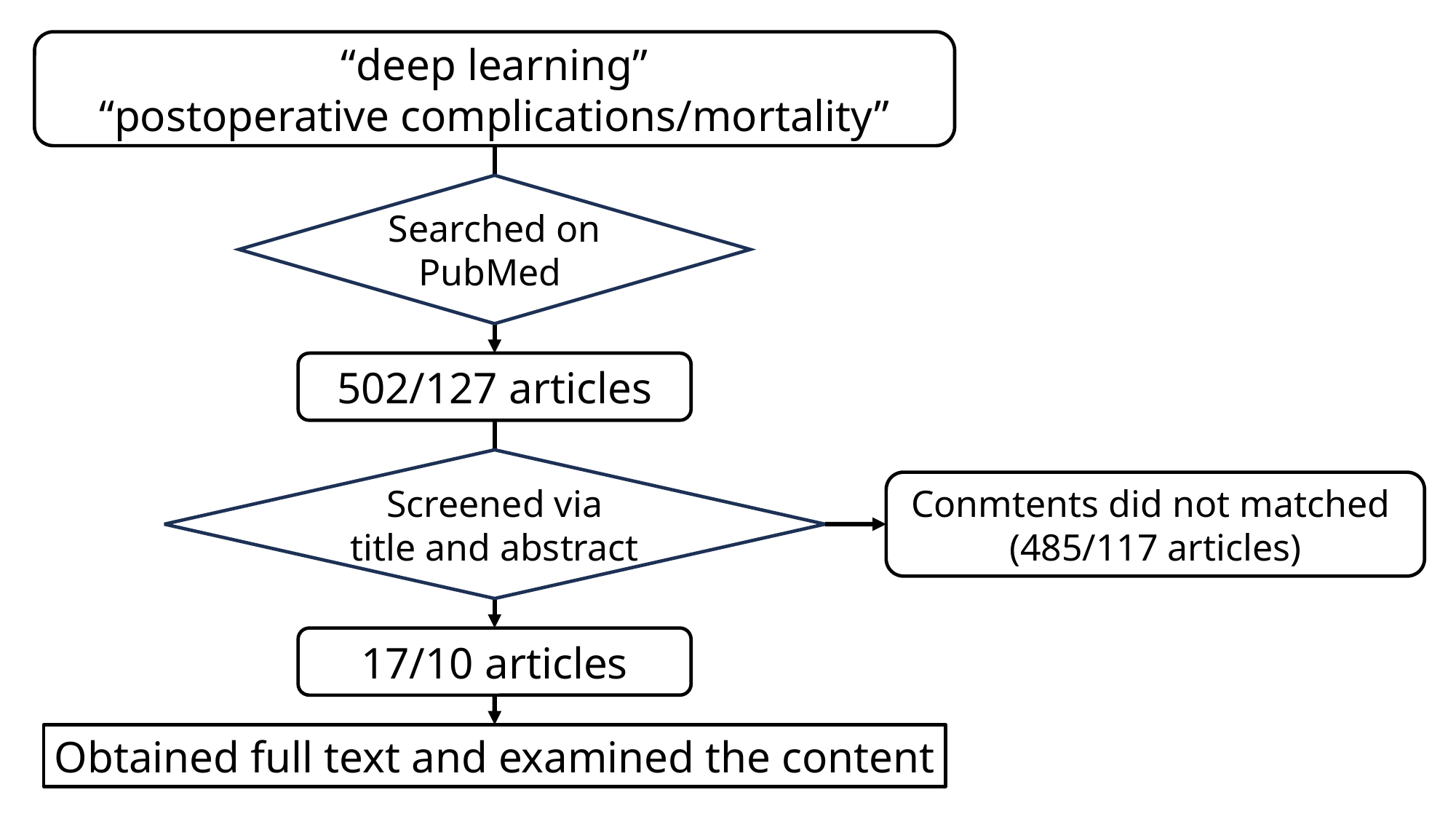

“deep learning”
“postoperative complications/mortality”
Searched on PubMed
502/127 articles
Screened via
title and abstract
Conmtents did not matched
(485/117 articles)
17/10 articles
Obtained full text and examined the content
